# A Dietary Assessment Training Course Path: The Italian IV SCAI Study on Children Food Consumption

**DOI:** 10.3389/fpubh.2021.590315

**Published:** 2021-03-12

**Authors:** Aida Turrini, Giovina Catasta, Laura Censi, Francisco Javier Comendador Azcarraga, Laura D'Addezio, Marika Ferrari, Cinzia Le Donne, Deborah Martone, Lorenza Mistura, Antonella Pettinelli, Raffaela Piccinelli, Anna Saba, Stefania Sette, Donatella Barbina, Debora Guerrera, Pietro Carbone, Alfonso Mazzaccara

**Affiliations:** ^1^Council for Agricultural Research and Economics, Research Centre for Food and Nutrition, Rome, Italy; ^2^Servizio Formazione – Presidenza, National Institute of Health, Rome, Italy

**Keywords:** professional community, dietary assessment, e-learning, training methods, innovative process

## Abstract

The eating patterns in a population can be estimated through dietary surveys in which open-ended assessment methods, such as diaries and interviews, or semi-quantitative food frequency questionnaires are administered. A harmonized dietary survey methodology, together with a standardized operational procedure, in conducting the study is crucial to ensure the comparability of the results and the accuracy of information, thus reducing uncertainty and increasing the reliability of the results. Dietary patterns (i) include several target variables (foods, energy and nutrients, other food components), (ii) require several explanatory variables (age, gender, anthropometric measurements, socio-cultural and economic characteristics, lifestyle, preferences, attitudes, beliefs, organization of food-related activities, etc.), and (iii) have impacts in several domains: imbalance diets; acute and chronic exposures affect health, specifically non-communicable diseases; and then sanitary expenditure. On the other hand, food demand has impacts on the food system: production, distribution, and food services system; food wastes and other wastes generated by food-related activities of the households (e.g., packaging disposal) have consequences on the “health of the planet” which in turn can have effects on human health. Harmonization and standardization of measurement methods and procedures in such a complex context require an *ad hoc* structured information system made by databases (food nomenclatures, portion sizes, food atlas, recipes) and methodological tools (quantification methods, food coding systems, assessment of nutritional status, data processing to extrapolate what we consider validated dietary data). Establishing a community of professionals specialized in dietary data management could lead to build a surveillance system for monitoring eating habits in the short term, thus reducing costs, and to arrange a training re-training system. Creating and maintaining the dietary data managers community is challenging but possible. In this context, the cooperation between the CREA Research Centre for Food and Nutrition and the Italian National Health Institute (ISS) promoted and supported by the Italian Ministry of Health may represent a model of best practice that can ensure a continuous training for the professional community carrying out a nutritional study.

## Introduction

The aim of this study is to illustrate the training system created for standardizing a community of professionals to collect food consumption data for surveillance system.

“… a healthy diet can contribute to achieving the global targets on NCDs adopted by the Sixty-sixth World Health Assembly, including achieving a 25% relative reduction in premature mortality from NCDs by 2025” (1).

Health and nutrition policy requires a thoroughly structured information system in order to provide indicators illustrating the current situation (2–4). This allows for appraising nutritional requirements at population level (LARN), risks intake exposure (EFSA), and possibility to be prone to have a non-communicable disease (5).

Standardization is a pre-requisite to correctly use epidemiological data (6), as we know that dietary data are structurally subjected to imprecision from the food description to the quantification. The dietary technique in open-ended response structures (diaries, 24 h recall, diet history) is designed to prompt respondents to provide a comprehensive and detailed report for all foods and beverages consumed.

Conducting a dietary survey requires highly trained fieldworkers able to administer the survey forms in the correct way (7, 8). Training systems are aimed at enabling interviewers (recall, diet history), administering survey forms (food diaries, food frequency questionnaires), and/or complementary questionnaires (food propensity questionnaire, background, lifestyle, physical activity, nutrition knowledge), and/or duplicating diets (9).

Training is an essential part of the study design, and usually, it is performed before starting the fieldwork of data collection (9, 10).

So, the risk to not engage sufficient practicality in conducting standardized interviews (recall/administration of diaries) and/or to forget some notions could occur (11, 12).

Nowadays, helpful well-developed tools, such as NUTRITOOLS, have been implemented with the aim to support researchers in charge of carrying out dietary surveys (see https://www.nutritools.org/). However, these very useful tools alone cannot ensure standardized practicality in conducting interviews, an activity that requires fieldwork experience to be fully acquired.

Moreover, motivation is an important factor for fieldworkers in such a kind of study that is very time consuming and personal commitment is demanding, particularly in the recruitment of subjects accepting to participate in the study (in the present case parents/caregivers of children whose food intakes have been recorded in 2 non-consecutive days), and then standardization is crucial to guarantee the reliability of the collected data. Fieldworkers in studies lasting 1 year to incorporate seasonal variability could be prone to lose the skill.

According to the above considerations, in the fourth national individual food consumption survey (IV SCAI) currently in progress in Italy, a structured training path has been implemented with the aim of selecting highly motivated professionals (to minimize the drop-out rate) and creating a highly-skilled professional community also for future similar studies. Medical doctors, biologists, dietitians, and nutritionists have been engaged, on a voluntary basis, because of the specific competence. A second objective, but not less important, was to involve professionals that people trust. In fact, the acceptability of the investigation is one of the major obstacles to sampled citizens' adhesion to a study. This causes a self-selection (refuse and substitution in the sampling framework) that introduces a bias although the random approach is adopted.

The rationale behind the experience here described is that a community of professionals specialized in dietary data collection and management will allow for building a permanent surveillance system in the short term and may reduce costs, to arrange a training re-training system to maintain the specialization and to prepare the turnover within the community.

Such a training system should be constantly updated on the technological tools for collecting food consumption data currently developed, especially, but not limited to, web-based and smartphone-based applications [as an example, see (13, 14)].

Creating and maintaining the dietary data managers community is challenging but possible. The CREA Centre for Food and Nutrition (CREA Food and Nutrition) and the National Institute of Health (Istituto Superiore di Sanità—ISS) are here sharing the experience that can help to design supportive training system to build communities of professionals able to carry out dietary data surveillance systems generating data for health managers, food system actors, and policymakers.

## Concept

**Figure 1:**
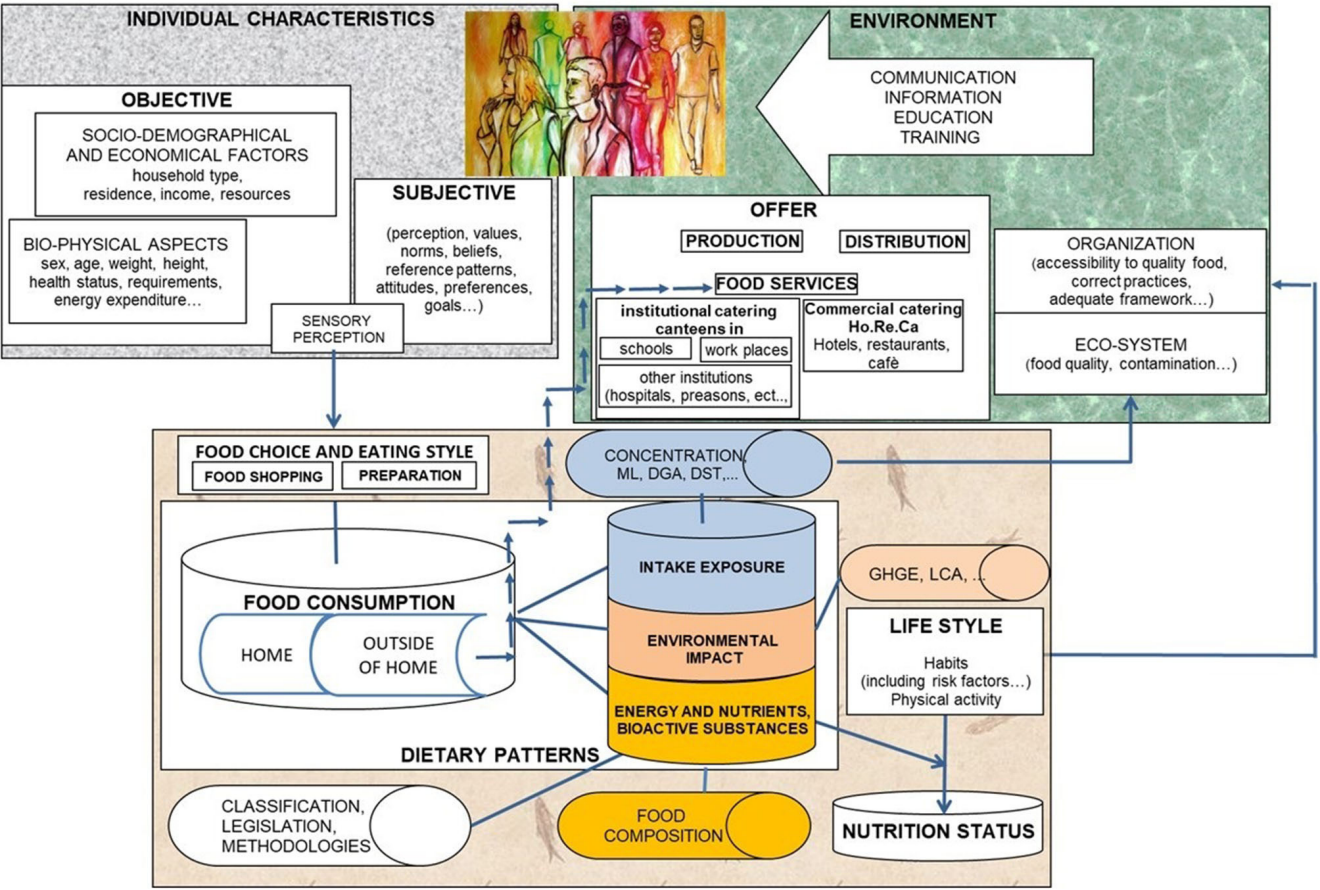
Conceptual scheme of applied research in nutrition—population studies.

The collection of individual food consumption from a nutritional point of view is a research activity in the field of population studies that have to do with the complexity, due to the number and the typology of factors that represent its determinants, the vast areas on which they have an impact, the adequacy of nutrient intake, exposure to the risk of taking undesirable substances, and environmental impact. The eating patterns of the population can be estimated through surveys in which diaries or interviews are administered or semi-quantitative frequency questionnaires. The choice of the methodology to apply for an observational study depends on factors of different natures: “scientific” or the type of data to be collected (diet history, eating habits, current consumption) linked, of course, to the objective of the study; “logistics” or the possibility and opportunity to carry out the study; and “economic” or the availability of resources—time, staff, and funds (9, 15). The specificity of the different age groups is an element considered essential for national studies, such as the pilot studies conducted for the activation of the EFSA EU-Menu program (10) have shown. A database system makes it possible to associate food composition tables, occurrences of contaminants, or greenhouse gas emission values with food consumption data. The alignment is carried out through a shared coding system (FoodEx2) (16). The data entry, management, and processing of data require the implementation of adequate software (10). The scientific context is summarized in Figure 1, which highlights the research activities and a set of possible interactions between different sectors of research on and for the consumer and, therefore, for all those interested in the information.

Complexity implies a high number of variables to be detected with the survey tools—quantity of foods consumed and their attributes, i.e., product, preparation and consumption mode, and processing, i.e., data entry, coding, aggregation, and calculation of derived variables, e.g., age, body mass index (BMI), basal metabolic rate (BMR), physical activity METs, health status, lifestyle, etc.

The variability of each variable is an intrinsic characteristic of the statistical variables. Precision and accuracy are essential to capture its variability and, therefore, estimate the distribution of each character and use them as input in multivariate analyses, trusting in the reliability of the measurements.

The sample design confers reliability on the study's ability to capture the variability and, therefore, the representativeness of the observations.

The calibration of the instruments ensures the accuracy of the measurements. It is a real calibration for analogical instruments, i.e., scales and stadiometers and infant meters, whereas the validation of the questionnaire and food data collection forms concerned the calibration of questionnaires and diaries and the modification of the software when necessary.

The preparation and standardization of the administration of the models tends to reduce the uncertainty deriving from the lack of accuracy that would confer an “artificial” variability, i.e., a variation between units observed that does not depend on the nature of the phenomenon, but on the effect due to the operator.

Therefore, the training of the interviewers is a basic element for the reliability of the data collected and thus the robustness of the estimates.

This concept is an extension of what in metrology is the search for measurement accuracy, in order to avoid the spread of error (17).

The training allows professionals who decide to dedicate themselves to a research activity to equip them with a knowledge base that allows them to correctly follow and manage the procedures for the implementation of the methodology envisaged in the project and acquire the related skills.

It is therefore necessary to include in the methodology provided for the training course in which the preparation is carried out, steps of training on an experiential basis, i.e., on-the-job training.

Since the mid-1990s, as the internet began to gather momentum, terms, such as computer-assisted learning, online learning, web-based learning, and e-learning, are often used synonymously, but all reflects knowledge transfer *via* an electronic device (18) and is now widely used in education.

Extensive systematic reviews and meta-analysis (19, 20), on the effectiveness of blended learning, highlighted that the participants were various health professional learners (nurses, medical students, nursing students, physicians, public health workers, and other categories) on a wide variety of health care disciplines, such as medicine, nursing, ethics, health policy, pharmacy, radiology, genetics, histology, and emergency preparedness. The results showed that between 1990 and 2019, none of the training courses were considered in the reviews, concerning the training of personnel specialized in the collection of food consumption data of the national population according to internationally approved guidelines.

**Figure 2:**
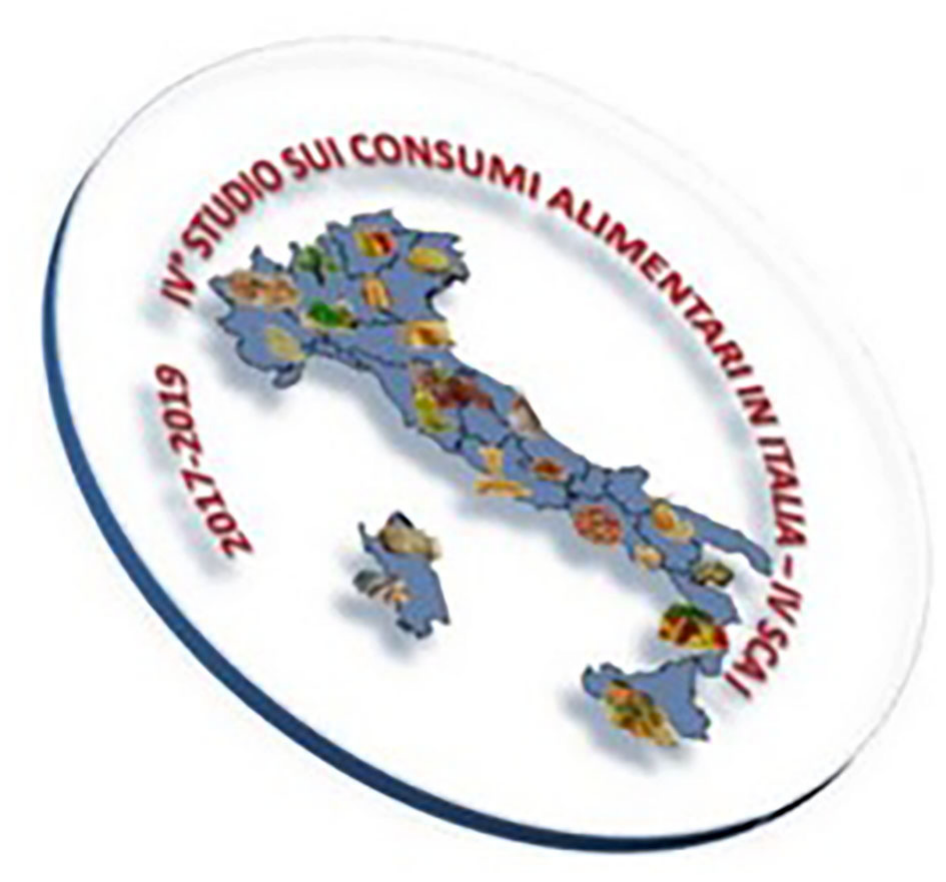
IV study on food consumption in Italy (IV SCAI).

Based on this consideration, to our knowledge, a training method performed as blended and hybrid learning course, i.e., “online and in-person classrooms,” and “multi-method, medium, and high interactive e-learning, on-the-job training” for highly specialized interviewers to collect data in the context of the IV Study on Food Consumption in Italy (Figure 2), for the cycle dedicated to food consumption of children from 3 months to 9 years on a national level, was the first to be implemented.

The training course duly modified in consideration of the different methodologies envisaged was replicated into three segments within the range of the 10–74 age group.

## Methodology

**Figure 3:**
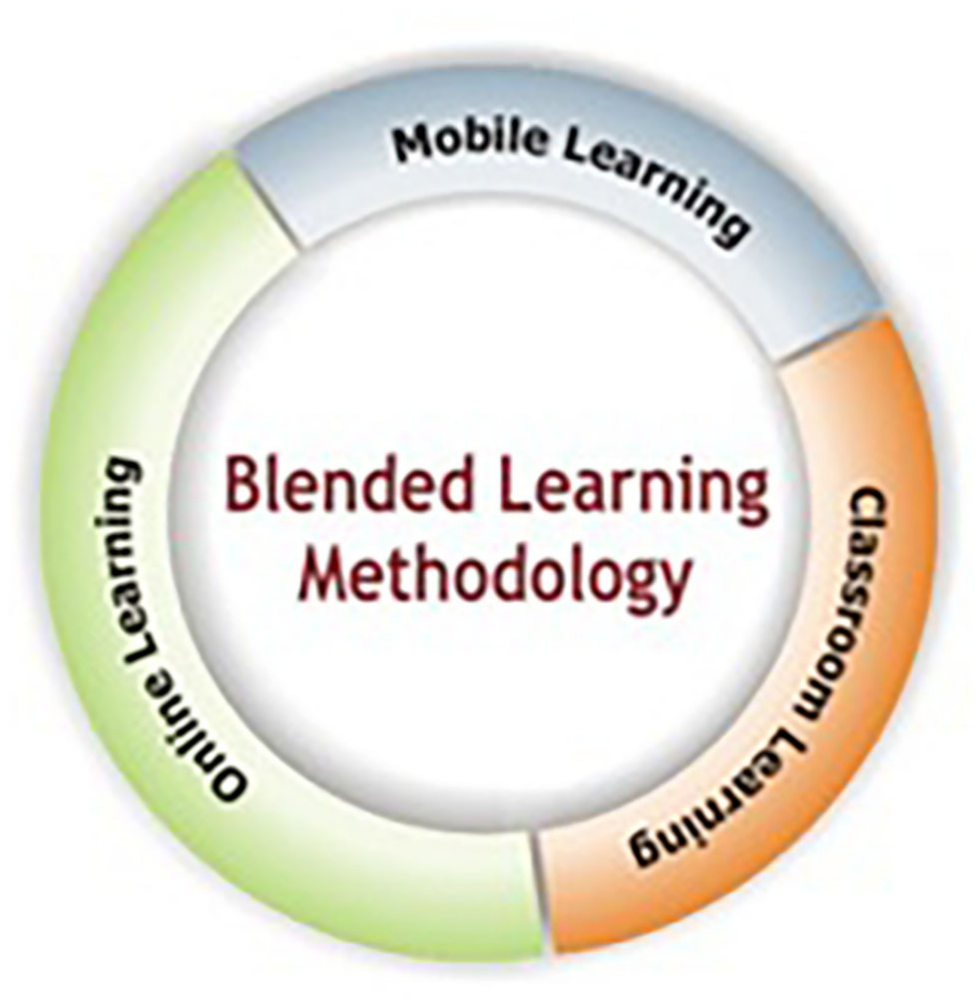
Blended e-learning model.

The approach chosen after extensive discussion and comparison between the partners of the training project—CREA-Food and Nutrition, Ministry of Health, National Institute of Health, is an approach defined as “blended” (Figure 3). In other words, a “model of mixed formation based on the set of formative moments in presence and at a distance. A typical blended learning course program involves alternating or the sequence of classroom meetings (lessons, seminars) and study phases, created using Distance Learning tools, whether they are CD-ROMs to be installed on your computer or real training courses available on the Internet in synchronous or asynchronous mode. It is currently believed that effective and comprehensive learning can be better achieved if it involves the simultaneous use of more strategies and more training tools. Therefore, real classrooms with face-to-face teachers and learners but also virtual classrooms, online tutors, network tutorship, books and paper handouts and advanced materials on CD-ROM. The complexity of a blended learning project requires the use of specific professional skills both at the didactic level (online training designers) and at the technological level” (Ebookecm.it).

The goal of the complete training course was to provide theoretical and practical knowledge to expert health professionals for the collection of food consumption in the age group from 3 months to 9 years at the national level, through the acquisition of specific skills on the use of tools and techniques for collecting food consumption necessary for carrying out a food survey according to the rules established at European level. So, a hybrid approach was adopted to guarantee a solid experiential after the theoretical training, i.e., medium interaction e-learning, residential, high interaction e-learning, and on-the-job research activity.

The teaching method adopted (first step) is inspired by the principles of Problem-Based Learning (PBL), where individual participants are activated by defining their own learning objectives and understanding and solving a problem, inspired by their professional context.

PBL is intended to increase knowledge and understanding by using appropriate problems that serve as a stimulus for learning (21, 22). Through the study of the didactic material selected by the experts and the search for further scientific material to achieve their learning objectives, the participant acquires new elements of knowledge and new skills for solving the problem itself. The questions at the end of the problem orient the learning process toward an applicative approach, and the keywords represent the stimulus for the autonomous research of the study material. In this way, the participant, urged to acquire knowledge and skills for the resolution of the case, is immediately the main architect of the learning process.

The hypothesis submitted to testing in this experience was the formation of the expert fieldworkers to properly carry out dietary surveys on a national scale. The successful vs. candidate participants' rate is a Key Performance Indicator (KPI). The collected data vs. total candidates (sensitivity) and vs. successful candidate (specificity) were then calculated.

## Results Achieved

**Figure 4:**
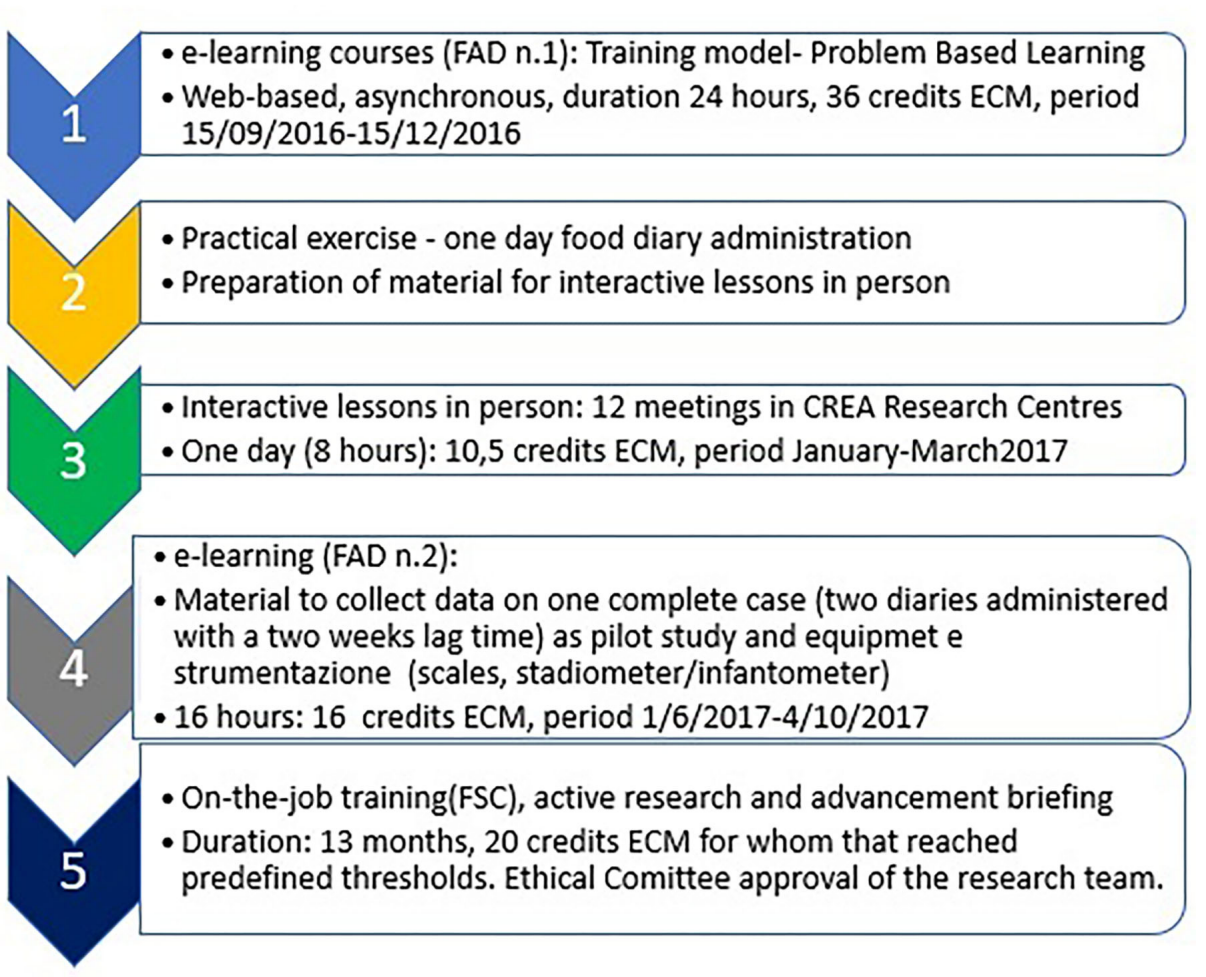
Flow chart course structure.

As the first result, the training course “National food consumption survey with harmonized methods according to the guidelines of the European Food Safety Authority (EFSA): theoretical and practical aspects, field activities and meaning in public health” is structured as illustrated in the flowchart shown in Figure 4. The training course was presented at the Ministry of Health during an event that was held on May 24, 2016, in which the health structures in the area were invited through the Prevention Departments, the Professional Registers, and the Scientific Societies that were welcomed to advertise with their subscribers that the first e-learning course within the blended path has been launched in 2016 (the list of interested organizations is reported in Appendix 1 in Supplementary Material).

The e-learning—period 15/09/2016–15/12/2016; registration until 30/11/2016; lessons: 24 h; ECM 36—course was structured into five parts, each with specific learning topics:

—first part—general information on food consumption surveys and on sample selection, calendar, and scheduling of the activities,
—second part—specific information concerning the recording of food consumption (food/beverages/supplements) using the food diary method,
—third part—information on the detection of anthropometric measurements and administer the socio-demographic questionnaire and the “Food Propensity Questionnaire,”
—fourth part—instructions on entering the consumption data recorded through the diary on a computer support, using the Foodsoft 1.0 software,^1^ and on performing the quality control of the data collected and entered,
—fifth part—the consequences for public health inherent in the use of the food consumption data collected.

Each of the eight tutors of the Training Course Team was a supervisor for one group, whereas a ninth tutor has been devoted to addressing anthropometric measurement questions.

The number of requests for enrolment in the first Distance Learning Course (DLC n.1) amounted to 779 vs. 500 eligible participants eligible to attend the first course candidate to receive Continuous Education in Medicine Credits (ECM^2^), credits ordered on the basis of a scoring system based on geographical criteria (balancing of areas), experience in the specific sector of nutritional surveys, the use of diet assessment tools, participation in national surveillance programs.

During the period of activity of the DLC n.1 course, 85 people who turned out to be inactive were replaced during the work until the timing calculated as sufficient to complete the required 24 h of learning. So, overall, 585 trainees attended the course, and then 276 successfully completed it and were invited to complete an exercise to use in the residential sessions.

Out of DLC n.1 trainees, 219 accepted to complete the exercise consisting in the compilation of a 1-day food diary (period 16/12/2016–15/01/2017), and 172 attended successfully one of the proposed in-person sessions-−12 interactive frontal lessons that were held in the CREA Centres throughout the national territory: Rome (three meetings), Casale Monferrato, Lodi (two meetings), Conegliano Veneto, Arezzo, Pontecagnano (two meetings), Bari, and Acireale.

The residential course program of 8 h (10.5 ECM) was based on checking the data collected during the exercise. Furthermore, the importance of some aspects of the survey of consumption was stressed, focusing attention on the basic points. In-person meetings had the main purpose to improve practicalities in conducting the data collection. Highly interactive sessions have been arranged.

The pilot study high interactive e-learning (DLC n.2) course (01/06/2017–04/12/2017; 16 h; 24 ECM) was attended and successfully completed by over 131 participants. All of them had been invited to attend the final on-the-job course (27/11/2017–29/06/2019; 20 ECM) participating in the research activity (Field Training—FT)^3^ consisting of data collection and data management (recruitment, data collection, data entry, data check, individual elaboration), and 119 accepted to attend the course.

Those who have completed *the whole course path* are highly motivated professionals and were 62 participants. The two questions “How do you evaluate the educational quality of the ECM program?” and “How do you evaluate the usefulness of this event for its training/updating?” obtained 100% positive evaluations, whereas the question “How do you assess the relevance of the topics covered in relation to your updating needs?” achieved 98%.

Considering the pilot study and the on-the-job courses, 812 valid cases had been collected. Therefore, the KPI “collected data vs. total candidates (sensitivity)” and “collected data vs. total candidates vs. successful candidate (specificity)” resulted in 812/500 = 1.4 and (812–131)/62 = 11.0 (131 cases of pilot studies are not here included as those had 100% successful cases).

Overall, the partial success rate (successes/enrollments) on the different phases of the course varies from 52.1% in the last phase (On-the-job-training-FT) to 100% in the DLC phase n. 2 interactive e-learning. However, the success rate of the entire course was 12.4%, as not all participants who completed the course agreed to enroll in the next phase with a withdrawal rate ranging from 0% (between physical exercises and residential session) to 23.8% (between residential session and DLC n. 2 interactive e-learning) (Table 1).

**Table 1:** Participation pattern described by enrolled, successful, accepted trainees at different phases.

**Course** ** number**	**Module**	**Enrolled**	**Successful/invited** ** to the next** ** phase**	**Accepted to** ** participate in the** ** subsequent course**	**Success** ** index**	**Withdraw** ** rate**
		**A**	**B**	**C**	**(B/A) %**	**[1-(C/B)]%**
1	DLC n. 1 e-learning	500	276	219	55.2%	20.7%
2	Exercise	219	172	172	78.5%	0.0%
3	Residential sessions	172	172	131	76.2%	23.8%
4	DLC n. 2 interactive e-learning	131	131	119	100.0%	9.2%
5	On-the-job training—FT	119	62		52.1%	

Considering the complexity of the issue, we estimate an average withdrawal rate of 13.3% from a course to another in the whole training path.

## Expected Impact

This process allows the formation of a community of highly specialized interviewers which can collect data on individual food consumption on a continuous basis. Community members are co-authors of scientific publications by citing the database.

A widened community will be established including all those who collaborated in various capacities in the study (mentioned in the acknowledgments of the publications), and indicating the role played (sharing of registry lists, administrative support, logistical support).

It will be necessary to ensure the training of new members and the updating for all and possibly organize themselves to provide European credits: European Credit Transfer and Accumulation System (ECTS).^4^

Ultimately, the project experiments with a model that can generate an infrastructure for research activities in the field of food and nutrition surveillance, equipped with an information system for assessing the total quality of the diet: nutritional adequacy, exposure to the risk of taking undesirable substances, and environmental impact. An open system for the introduction of future problem is expected to make easier to experiment with new survey methods that can reduce the burden for fieldworkers and participants, promoting an increase in the acceptance rate, while ensuring accuracy and precision.

Differences in implementing the 10–74 years old food consumption survey, mainly due to differences in methodology, but also related to learned lessons, will be discussed in a subsequent publication once the ongoing activity will be over.

In the long-term, the constitution of a specialized professional community to carry out individual food consumption surveys is expected to save public money mainly embedding the survey in the public health system according to the present experience.

In our experience, past surveys involving around 1,300 households (about 3,300 individuals) had an estimated cost of around 1 million euros, 1/3 devoted to the engagement of private companies, the rest consisting in material, functioning, and in-kind contribution of personnel engagement within the CREA Food and Nutrition^5^ [ref. individual food consumption survey carried out by Leclercq et al. (23)]. Therefore, at least costs for externalities can be saved and invested in other public health actions.

## Discussion

The main novelty of this work is that it was the first e-learning system in the EU developed with the aim of selecting highly motivated professionals and creating a highly-skilled professional community specialized in the collection of national food consumption data according to internationally approved guidelines.

A study on national food consumption requires a representative data collection of the population carried out throughout the territory. This includes the involvement of a sufficient number of interviewers. A training course structured as described in this work allowed to reach a large number of professionals at the same time and train them in a standardized and replicable way. We can say that one of the limitations encountered in the system devised by CREA was the organization of 12 interactive frontal lessons that were held in the CREA Centres throughout the national territory. This part of the course involved a movement of people (tutors and participants) and tools for the execution of the meetings, causing an increase in costs in terms of both time and money. This confirmed that an e-learning course is more flexible and manageable for the student, whereas the residential course has the limitation of the face-to-face meetings, and the students have to dedicate 2 prefix days for following the course.

The rationale for adopting a blended courses path was to monitor the deep knowledge acquired in the e-learning and residential phases, and the consolidated practicality in the pilot phases (second interactive e-learning) by the interviewers was maintained along the whole survey period to ensure the highest level possible of collected data quality.

The main limitation of the present work is represented by the lack of previous similar experiences to estimate the effectiveness of the approach.

We hope that future nationwide individual dietary surveys will gain from the results here obtained to plan a continuous surveillance nutritional system.

Another limitation could be the sustainability of the e-learning course because it always entails management costs.

Adjustments on practical implementation will be necessary to take into account the utilization of new technologies [someone among several (6, 13)]. The role of interviewers can be shifted to data management, as an example, moving the activity from interviewing to interacting with the data provider to check and clean.

The need for setting up a system where the professional community represents the core to be periodically renewed, to refresh and update the knowledge, to replace persons leaving the community, and to expand the group according to the needs of data is a must.

Coordination among public institutions involved in the surveillance system is a crucial aspect. In the present experience, three public bodies have been involved (CREA, Ministry of Health, National Institute of Health), but considering the high interest in dietary data for the whole food system, the engagement of other public bodies is desirable.

The Italian law encourages the collaboration between public institutions to optimize the use of resources, avoiding duplications of expenditure.^6^

This can lead to further beneficial effects, particularly the reduction of expenditures related to the economic cost of unhealthy diets [see e.g., (24)]. Literature reports examples of needs for training courses for the general public and stakeholders in the nutrition and food system field [as an example, see (25, 26)]. This is in line with the healthy and sustainable diets issue widely treated in the Willett et al. (26) publication. However, a community sharing interests, motivation, and expertise plays a role in pushing toward a sustainable monitoring system that is expected to provide indicators for policymakers, local authorities, and other stakeholders.

## Conclusion

Creating and maintaining the community of dietary data managers is challenging but possible. CREA Food and Nutrition and the Italian National Health Institute (ISS) teams that implemented and managed the course path here proposed are sharing the experience that can help to design supportive training system to build communities of professionals able to carry out dietary data surveillance systems generating data for health managers, food system actors, local authorities, and policymakers. Such a kind of a synergistic approach will help to save public money because of the optimization of resources available in the Public Administration to produce the necessary information basis. The collaboration between CREA and ISS promoted and supported by the Italian Ministry of Health is a best practice example of public institutions collaboration the authors consider very fruitful and worth to be replicated in future occasions, especially in setting up a nutritional surveillance system also in complementarity with other current monitoring activities on health. This is considered particularly helpful in the coronavirus disease 2019 (COVID-19) pandemic situation is crucial to face unexpected outbreaks.

## Data Availability Statement

The original contributions presented in the study are included in the article/Supplementary Material, further inquiries can be directed to the corresponding author/s.

## Ethics Statement

The studies involving human participants were reviewed and approved by Comitato Etico Indipendente Lazio 2, Lazio Region (Italy). Written informed consent to participate in this study was provided by the participants' legal guardian/next of kin.

## Author Contributions

AT has proposed and developed the concept together with GC, LC, FJCA, LD'A, MF, CLD, DM, LM, AP, RP, AS, SS, DB, DG, PC, and AM, has prepared the first draft and all subsequent versions. GC, LC, FJCA, LD'A, MF, CLD, DM, LM, AP, RP, AS, SS, DB, DG, PC, and AM have revised and approved all versions. GC, FJCA, LD'A, MF, CLD, DM, LM, AP, RP, and SS have developed the concept, prepared all the material for the courses path, tutored participants, and managed the survey in all the phases. LC has developed and supported the part of the course devoted to anthropometric measurements. AS has supported all the parts concerning the consumer's science. AM has coordinated and co-designed the concept of the e-learning course implemented and supported by DB, DG, and PC that have all supported the implementation of the e-learning courses.

## Training Course Team

Training Course Team of the IV SCAI training path (3months−9 years old and 10–74 years old food consumption survey conduction according to the EU-Menu EFSA methodology).

### CREA Food and Nutrition

Course project staff: Aida Turrini (responsible for scientific data, course director), Giovina Catasta, Laura Censi, Laura D'Addezio, Marika Ferrari, Cinzia Le Donne, Deborah Martone, Lorenza Mistura, Raffaela Piccinelli, Anna Saba, and Stefania Sette.

Teachers/Tutors: Giovina Catasta, Laura Censi, Donatella Ciarapica, Francisco Javier Comendador Azcarraga, Laura D'Addezio, Alessandra Durazzo, Marika Ferrari, Cinzia Le Donne, Deborah Martone, Lorenza Mistura, Raffaela Piccinelli, Laura Rossi, Anna Saba, Stefania Sette, Elisabetta Toti, and Dario Berardi (Dev4U Srl).

Scientific Secretariat: Cinzia Le Donne, Raffaela Piccinelli, and Stefania Sette.

Management Secretariat: Antonella Pettinelli.

### National Institute of Health

Course project staff: Donatella Barbina, Debora Guerrera, Alfonso Mazzaccara, Pietro Carbone, and Federica Maria Regini.

Course Director: Umberto Agrimi.

Teachers: Francesco Cubadda and Marco Silano.

Scientific Secretariat: Francesco Cubadda and Marco Silano.

Management Secretariat: Emiliana Falcone and Valeria Patriarca.

### Ministry of Health

Course Director: Rossana Valentini.

Scientific Secretariat: Francesca Calvetti, Delia Forte, and Roberto Copparoni.

## Conflict of Interest

The authors declare that the research was conducted in the absence of any commercial or financial relationships that could be construed as a potential conflict of interest.

## Footnotes

^1^Website URL: https://creafad.dev4u.it/.

^2^Italian acronym ECM=Educazione Continua in Medicina.

^3^Italian acronym FSC=Formazione Sul Campo (on-the-job training research activity).

^4^http://ec.europa.eu/education/resources/european-credit-transfer-~accumulation-system_en.

^5^Former INRAN, Italian National Institute for Food and Nutrition Research.

^6^https://www.codiceappalti.it/l_241_1990/art__15_(accordi_fra_pubbliche_amministrazioni)/1566 [translation by the authors: procurement code l. 241/1990/art, 15 (agreements between public administrations)].

## References

[1] World Health Organization. Vienna Declaration on Nutrition and Noncommunicable Diseases in the Context of Health. 2020. WHO Ministerial Conference on Nutrition and Noncommunicable Diseases in the Context of Health 2020. (2013). Available online at: https://www.euro.who.int/__data/assets/pdf_file/0003/234381/Vienna-Declaration-on-Nutrition-and-Noncommunicable-Diseases-in-the-Context-of-Health-2020-Eng.pdf

[2] WHO. Framework and Standards for Country Health Information Systems. (2008). Available online at: https://www.who.int/healthinfo/country_monitoring_evaluation/who-hmn-framework-standards-chi.pdf

[3] World Health Organization Regional Office for the Eastern Mediterranean Al Jawaldeh A Osman D Tawfik A. Food and Nutrition Surveillance Systems: A Manual for Policy-Makers and Programme Managers? (2014). Available online at: https://apps.who.int/iris/handle/10665/259796

[4] WHO. Integrated Surveillance of Noncommunicable Diseases (iNCD). (2015). Available online at: https://ec.europa.eu/health/sites/health/files/indicators/docs/incd_en.pdf

[5] Global Burden of Disease 2015 Risk Factors Collaborators. Global, regional, and national comparative risk assessment of 79 behavioural, environmental and occupational, and metabolic risks or clusters of risks, 1990–2015: a systematic analysis for the Global Burden of Disease Study 2015. Lancet. (2015) 388:1659–724. 10.1016/S0140-6736(16)31679-8 PMC5388856 27733284

[6] Turrini A D'Addezio L Dhurandhar E Ferrari M Le Donne C Mistura L. Editorial: emerging topics in dietary assessment. Front Nutr. (2019) 19:176. 10.3389/fnut.2019.00176 31803751 PMC6877603

[7] Willet W. Nutritional epidemiology: issues and challenges. Int J Epidemiol. (1987) 16:312–7. 10.1093/ije/16.2.312 3610460

[8] Cade JE Warthon-Medina M Albar S Alwan NA Ness A Roe M. DIET@NET: best practice guidelines for dietary assessment in health research. BMC Med. (2017) 15:202. 10.1186/s12916-017-0962-x 29137630 PMC5686956

[9] Food and Agriculture Organization of the United Nations. Dietary Assessment: A Resource Guide to Method Selection and Application in Low Resource Settings. Rome: FAO (2018). Available online at: http://www.fao.org/3/i9940en/I9940EN.pdf

[10] European Food Safety Authority. Guidance on the EU menu methodology. EFSA J. (2014) 12:3944. 10.2903/j.efsa.2014.3944

[11] Committee on Dietary Risk Assessment in the WIC Program. Dietary Risk Assessment in the WIC Program. Food and Nutrition Board (2002). Washington, DC: The National Academies Press. 10.17226/10342

[12] Dao MC Subar AF Warthon-Medina M Cade JE Burrows T Golley RK. Dietary assessment toolkits: an overview. Public Health Nutr. (2019) 22:404–18. 10.1017/S1368980018002951 30428939 PMC6368251

[13] Doulah TG Hossain D Imtiaz MH Sazonov E. “Automatic ingestion monitor version 2” – a novel wearable device for automatic food intake detection and passive capture of food images. IEEE J Biomed Health Inform. (2021) 25:568–76. 10.1109/JBHI.2020.2995473 32750904 PMC7938421

[14] Tay W Kaur B Quek R Lim J Henry CJ. Current developments in digital quantitative volume estimation for the optimisation of dietary assessment. Nutrients. (2020) 12:1167. 10.3390/nu12041167 32331262 PMC7231293

[15] Turrini A. Indagini alimentari su scala nazionale: metodologia e possibilità di utilizzazione. Giornale Eur Nutr Clin. (1993) 3:61–9.

[16] European Food Safety Authority. The Food Classification and Description System FoodEx2 (Revision 2). EFSA Supporting Publication 2015:EN-804 (2015). Available online at: http://www.efsa.europa.eu/sites/default/files/assets/804e.pdf

[17] Turrini A. Looking at metrological concepts in dietary assessment. In: Proceedings of the 2nd IMEKOFOODS “Promoting Objective and Measurable Food Quality & Safety”, October 2nd−5th 2016, Benevento (2016).

[18] Sinclair P Kable A Levett-Jones T. The effectiveness of internet-based e-learning on clinician behavior and patient outcomes: a systematic review protocol. JBI Database Syst Rev Implement Rep. (2015) 13:52–64. 10.11124/jbisrir-2015-1919 26447007

[19] Liu Q Peng W Zhang F Hu R Li Y Yan W. The effectiveness of blended learning in health professions: systematic review and meta-analysis. J Med Internet Res. (2016) 18:e2. 10.2196/jmir.4807 26729058 PMC4717286

[20] Vallée A Blacher J Cariou A Emmanuel S. Blended learning compared to traditional learning in medical education: systematic review and meta-analysis. J Med Internet Res. (2020) 22:e16504. 10.2196/16504 32773378 PMC7445617

[21] Wood DF. Problem based learning. BMJ. (2003) 326:328–30. 10.1136/bmj.326.7384.328 12574050 PMC1125189

[22] Hinneburg J Lühnen J Steckelberg A Berger-Höger B. A blended learning training programme for health information providers to enhance implementation of the Guideline Evidence-based Health Information: development and qualitative pilot study. BMC Med Educ. (2020) 20:77. 10.1186/s12909-020-1966-3 32183798 PMC7079382

[23] Leclercq C Arcella D Piccinelli R Sette S Le Donne C Turrini A. The Italian National Food Consumption Survey INRAN-SCAI 2005-06. Main results in terms of food consumption. Public Health Nutr. (2009) 12:2504–32. 10.1017/S1368980009005035 19278564

[24] Candari CG Cylus J Nolte E. Assessing the Economic Costs of Unhealthy Diets and Low Physical Activity: An Evidence Review and Proposed Framework. European Commission, Observatory on Health System and Policies. Health Policy Series 47. World Health Organization 2017 (Acting as the Host Organization for, and Secretariat of, the European Observatory on Health Systems and Policies), NCBI Bookshelf (2017). 28787114

[25] Food and Agriculture Organization of the United Nations. Nutrition and Food System. HLPE–High Level Panel of Experts Series. Rome: FAO (2017). Available online at: http://www.fao.org/3/a-i7846e.pdf

[26] Willett W Rockström J Loken B Springmann B Lang T Vermeulen S. Food in the anthropocene: the EAT-Lancet Commission on healthy diets from sustainable food systems. Lancet Comm. (2019) 393:447–92. 10.1016/S0140-6736(18)31788-4 30660336

