# Corrigendum: A Dietary Assessment Training Course Path: The Italian IV SCAI Study on Children Food Consumption

**DOI:** 10.3389/fpubh.2021.708291

**Published:** 2021-06-09

**Authors:** Aida Turrini, Giovina Catasta, Laura Censi, Francisco Javier Comendador Azcarraga, Laura D'Addezio, Marika Ferrari, Cinzia Le Donne, Deborah Martone, Lorenza Mistura, Antonella Pettinelli, Raffaela Piccinelli, Anna Saba, Stefania Sette, Donatella Barbina, Debora Guerrera, Pietro Carbone, Alfonso Mazzaccara

**Affiliations:** ^1^Council for Agricultural Research and Economics, Research Centre for Food and Nutrition, Rome, Italy; ^2^Servizio Formazione–Presidenza, National Institute of Health, Rome, Italy

**Keywords:** professional community, dietary assessment, e-learning, training methods, innovative process

In the original article, there was an error in the acknowledgments, as **the name of “Eliane Denis Bahbouth” was missing**.

A correction has been made to ^**^Acknowledgment^**^

^**^Insert CORRECTED paragraph^**^

The authors thank Dr. Gaetana Ferri (Ministry of Health) for her enthusiastic support and Dr. Elisabetta Lupotto (CREA Food and Nutrition) for her supportive commitment. Our immense gratitude goes to all participants for their great and competent commitment in completing the IV SCAI 3 months−9 years old individuals: Rosaria Amabile, Cristina Baggio, **Eliane Denise Bahbouth**, Lauretta Bianco, Lucia Bonadies, Filomena Capasso, Sabrina Capineri, Rosa Carbone, Roberta Carli, Liliana Cassano, Maurizio Cavallaro, Rosalba Cipresso, Stefania Corradi, Alessandra Covino, Maria Cristina Cucugliato, Sara Dattoli, Maria De Marinis, Federica Del Genio, Flora Di Tommaso, Emanuela Alessandra Donghi, Roberta Falcone, Federica Falvo, Elena Felloni, Anna Ferrante, Antonella Foglia, Paola Golzio, Silvana Grasso, Daniele Grumiro, Emilia Guberti, Marina La Rocca, Patrizia Lamberti, Elisa Lazzarino, Rosanna Macaluso, Manuela Maione, Lara Marangoni, Maria Rita Milone, Antonio Molinaro, Erika Mollo, Laura Morisi, Giuseppa Pacella, Michele Parmato, Angela Pasinato, Brunella Pasquini, Franca Pasticci, Emma Petrella, Carolina Poli, Luigi Polverino, Paola Pozzo, Lisa Randisi, Valeria Rebonato, Vittoria Rocchino, Monica Ruotolo, Angela Silvestri, Gabriella Siniscalchi, Susanna Tardonato, Paola Tei, Francesca Trinchella, Silvia Tulone, Salvatore Vaccaro, Anna Valente, Lorella Vicari, and Sara Vignozzi. The training course for the training of highly specialized interviewers for the IV SCAI study was developed within the EU-Menu program of EFSA; the implementation of the innovative training model was carried out with the support of the Determinants of Diet and Physical Activity Knowledge Hub (DEDIPAC) project, as part of the Joint Programming Initiative (JPI) A Healthy Diet for a Healthy Life (HDHL), which for Italy was led by the IRILD consortium Research support infrastructures to promote an active lifestyle and a healthy diet.

The authors apologize for this error and state that this does not change the scientific conclusions of the article in any way. The original article has been updated.

