# Supplementary material for: A Dietary Assessment Training Course Path: The Italian IV SCAI Study on Children Food Consumption
Source: Front Public Health. 2021 Mar 12;9:590315. doi: 10.3389/fpubh.2021.590315 (PMC7994926; doi:10.3389/fpubh.2021.590315)
Supplement: Supplementary file 1 [file Data_Sheet_1.PDF]

Appendix 1 - Candidates to participate in the Continue Education in Medicine (ECM) Course "Food Consumption": number of questions for membership in Orders-Society-Associations (OSA) (multiple answers possible)

Main group	Society/registered Professionals	Acronym	Website	Candidates number	September 2016 %	English name
	Ordine Nazionale dei Biologi	ONB	http://www.onb.it/	257	32,6%	National Order of Biologists
	Associazione Italiana Dietisti	ANDID	http://www.andid.it/	129	16,4%	Italian Dietitians Association
	Ordine dei Medici Chirurghi e degli Odontoiatri di provincia	OMCEOxx	www.omceo.XX.it	124	15,7%	Order of Medical Surgeons and Dentists of the province
	Federazione Italiana Medici Pediatri	FIMP	http://www.fimp.pro/	40	5,1%	Italian Federation of Pediatricians
	Associazione Italiana di Dietetica e Nutrizione Clinica	ADI	http://www.adiitalia.net/	29	3,7%	Italian Association of Dietetics and Clinical Nutrition
	Società Italiana di Nutrizione Umana	SINU	http://www.sinu.it/	28	3,6%	Italian Society of Human Nutrition
	Società Italiana di Igiene Medicina Preventiva e Sanità Pubblica	SITI	http://www.societaitalianaigiene.org/site/new/	22	2,8%	Italian Society of Hygiene, Preventive Medicine and Public Health
	Società Italiana Medici Pediatri	SIMPe	http://www.simpe.it/	17	2,2%	Italian Society of Pediatricians
FIMMG	Società Italiana di Medicina di Prevenzione e degli Stili di Vita	SIMPESV	http://alimentazione.fimmg.org/	16	2,0%	Italian Society of Prevention and Lifestyles Medicine
	Società Italiana di Pediatria	SIP	http://sip.it/	15	1,9%	Italian Society of Pediatrics
	Federazione Italiana Medici di Famiglia	FIMMG	http://www.fimmg.org/	14	1,8%	Italian Federation of Family Doctors
	Società Italiana di Medicina Generale e delle cure primarie	SIMG	https://www.simg.it/	12	1,5%	Italian Society of General Medicine and Primary Care
	Società Italiana di Nutrizione Clinica e Metabolismo	SINUC	http://www.sinuc.it/index.php	12	1,5%	Italian Society of Clinical Nutrition and Metabolism
	Società Italiana di Nutrizione artificiale e metabolismo	SINPE	http://www.sinpe.org/	8	1,0%	Italian Society of Artificial Nutrition and Metabolism
	Associazione Culturale Pediatri	ACP	http://www.acp.it/	6	0,8%	Cultural Association of Pediatricians
	Federazione Nazionale degli ordini dei Medici Chirughi e degli Odontoiatri	FNOMCEO	https://portale.fnomceo.it/fnomceo/home.2puntOT	5	0,6%	National Federation of the orders of Chirughi Doctors and Dentists
	Società Italiana dell'Obesità	SIO	http://sio-obesita.org/	5	0,6%	Italian Obesity Society
	Società Scientifica di Nutrizione Vegetariana	SSNV	http://www.scienzavegetariana.it/	4	0,5%	Scientific Society of Vegetarian Nutrition
	Ordine dei Tecnologi Alimentari Campania e Lazio	OTACL	http://www.otacl.gov.it/	3	0,4%	Order of Food Technologists Campania and Lazio
SIP	Società Italiana di Medicina Emergenza Urgenza Pediatrica	SIMEUP	http://www.simeup.it/	3	0,4%	Italian Society of Pediatric Emergency Medicine
	Società Italiana per lo Studio dei Disturbi del Comportamento Alimentare	SISDCA	http://sisdcadisturbialimentari.weebly.com/	3	0,4%	Italian Society for the Study of Eating Disorders
	Associazione Biologi Nutrizionisti Italiani	ABNI	http://www.abni.it/	2	0,3%	Association of Italian Nutrition Biologists
	Associazione Italiana Nutrizionisti	AINUT	http://www.ainut.it/	2	0,3%	Italian Association of Nutritionists
	Associazione onlus Nutrizionisti senza Frontiere	NUSEF	http://www.nutrizionistisenzafrontiere.org/	2	0,3%	Non-profit Association Nutritionists Without Borders
	Società Italiana delle Cure Primarie Pediatriche	SICUPP	http://www.sicupp.org/	2	0,3%	Italian Society of Pediatric Primary Care
	Società Italiana di Allergologia e Immunologia Pediatrica	SIAIP	http://www.siaip.it/	2	0,3%	Italian Society of Pediatric Allergy and Immunology
	Società Italiana di Chirurgia dell'Obesità e delle malattie metaboliche	SICOB	http://www.sicob.org/	2	0,3%	Italian Society of Obesity Surgery and Metabolic Diseases
	Società Italiana di Diabetologia	SID	http://www.siditalia.it/	2	0,3%	Italian Society of Diabetology
	Società Italiana di Pediatria Preventiva e Sociale	SIPPS	http://www.sipps.it/	2	0,3%	Italian Society of Preventive and Social Pediatrics
	The European Society for Clinical Nutrition and Metabolism	ESPEN	http://www.espen.org/	2	0,3%	The European Society for Clinical Nutrition and Metabolism
	Associazione Italiana Disturbi dell'Alimentazione e del Peso	AIDAP	http://aidap.org/	1	0,1%	Italian Association of Eating and Weight Disorders
	Associazione Nazionale Specialisti in Scienza dell'Alimentazione	ANSISA	http://www.ansisa.it/	1	0,1%	National Association of Specialists in Food Science
	European Academy of Paediatrics	EAP	www.eapaediatrics.eu	1	0,1%	European Academy of Paediatrics
	Fondazione Dieta Mediterranea	FONDAZIONE DIETA MEDITERRANEA	http://www.fondazionedietamediterranea.it/	1	0,1%	Mediterranean Diet Foundation
	FONDAZIONE ITALIANA PER L'EDUCAZIONE ALIMENTARE	FOODEDU	http://www.foodedu.it/en/p	1	0,1%	ITALIAN FOUNDATION FOR FOOD EDUCATION
ANDID	Gruppo Italiano di Cardiologia Riabilitativa e Preventiva	GICR	http://www.gicr.it/index.php?p=44	1	0,1%	Italian Group of Rehabilitation and Preventive Cardiology
	Società Italiana di Allergologia, Asma e Immunologia Clinica	SIAAIC	http://www.siaaic.eu/	1	0,1%	Italian Society of Allergy, Asthma and Clinical Immunology
	Società Italiana di Biochimica Clinica e Biologia Molecolare Clinica	SIBIOC	http://www.sibioc.it/	1	0,1%	Italian Society of Clinical Biochemistry and Clinical Molecular Biology
	Società Italiana di Medicina delle Migrazioni	SIMM	http://www.simmweb.it/	1	0,1%	Italian Society of Migration Medicine
	Società Italiana di Medicina Funzionale	SIMF	http://www.simf.it/it/#	1	0,1%	Italian Society of Functional Medicine
	Società Italiana di Nutraceutica	SINUT	http://www.sinut.it/	1	0,1%	Italian Society of Nutraceuticals
SIP	Società Italiana di Nutrizione Pediatrica	SINUPE	http://sip.it/pianeta-sip/societa-affiliate/sinupe-societa-italiana-di-nutrizione-pediatrica	1	0,1%	Italian Society of Pediatric Nutrition
	Società Italiana di Patologia Clinica e Mediciona di Laboratorio	SIPMEL	http://www.sipmel.it/it/	1	0,1%	Italian Society of Clinical Pathology and Laboratory Medicine
	Società Italiana di Pediatria Ospedaliera	SIPO	http://www.pediatriaospedaliera.org/	1	0,1%	Italian Society of Hospital Pediatrics
	Società Italiana di Riabilitazione Interdisciplinare Disturbi Alimentari e del Peso	SIRIDAP	http://www.siridap.org/	1	0,1%	Italian Society of Interdisciplinary Rehabilitation Eating and Weight Disorders
	Società Italiana Nutizione Sport e Benessere	SINSEB	http://www.sinseb.it/	1	0,1%	Italian Society of Nutrition, Sport and Wellness
	Società Italiana per lo Studio dell'Emostasi e della Trombosi	SISET	http://www.siset.org/	1	0,1%	Italian Society for the Study of Haemostasis and Thrombosis
FIMMG	Società Scientifica dei Medici di Medicina Generale	METIS	http://fimmg.org/index.php?action=pages&m=view&p=280	1	0,1%	Scientific Society of General Practitioners
